# Polyfunctional anti-human epidermal growth factor receptor 3 (anti-HER3) antibodies induced by HER3 vaccines have multiple mechanisms of antitumor activity against therapy resistant and triple negative breast cancers

**DOI:** 10.1186/s13058-018-1023-x

**Published:** 2018-08-09

**Authors:** Takuya Osada, Zachary C. Hartman, Junping Wei, Gangjun Lei, Amy C. Hobeika, William R. Gwin, Marcio A. Diniz, Neil Spector, Timothy M. Clay, Wei Chen, Michael A. Morse, H. Kim Lyerly

**Affiliations:** 1 Division of Surgical Sciences, Department of Surgery, Duke University Medical Center, MSRB Research Drive, Box 2714, Durham, NC 27710 USA; 2 Division of Medical Oncology, Department of Medicine, University of Washington, Seattle, WA USA; 3 Biostatistics and Bioinformatics Research Center, Samuel Oschin Comprehensive Cancer Institute, Cedars-Sinai Medical Center, Los Angeles, CA USA; 4 Division of Medical Oncology, Department of Medicine, Duke University Medical Center, Durham, NC USA; 5 Cell and Gene Therapy Discovery Research, PTS, GlaxoSmithKline, Collegeville, PA USA; 6 Division of Gastroenterology, Department of Medicine, Duke University Medical Center, Durham, NC USA; 7 Division of General Surgery, Department of Surgery, Duke University Medical Center, Durham, NC USA

**Keywords:** HER3, HER2, Immunotherapy, Adenovirus, Polyclonal antibodies, ErbB3

## Abstract

**Background:**

Upregulation of human epidermal growth factor receptor 3 (HER3) is a major mechanism of acquired resistance to therapies targeting its heterodimerization partners epidermal growth factor receptor (EGFR) and human epidermal growth factor receptor 2 (HER2), but also exposes HER3 as a target for immune attack. We generated an adenovirus encoding full length human HER3 (Ad-HER3) to serve as a cancer vaccine. Previously we reported the anti-tumor efficacy and function of the T cell response to this vaccine. We now provide a detailed assessment of the antitumor efficacy and functional mechanisms of the HER3 vaccine-induced antibodies (HER3-VIAs) in serum from mice immunized with Ad-HER3.

**Methods:**

Serum containing HER3-VIA was tested in complement-dependent cytotoxicity (CDC) and antibody-dependent cellular cytotoxicity (ADCC) assays and for its effect on HER3 internalization and degradation, downstream signaling of HER3 heterodimers and growth of metastatic HER2+ (BT474M1), HER2 therapy-resistant (rBT474), and triple negative (MDA-MB-468) breast cancers.

**Results:**

HER3-VIAs mediated CDC and ADCC, HER3 internalization, interruption of HER3 heterodimer-driven tumor signaling pathways, and anti-proliferative effects against HER2+ tumor cells in vitro and significant antitumor effects against metastatic HER2+ BT474M1, treatment refractory HER2+ rBT474 and triple negative MDA-MB-468 in vivo.

**Conclusions:**

In addition to the T cell anti-tumor response induced by Ad-HER3, the HER3-VIAs provide additional functions to eliminate tumors in which HER3 signaling mediates aggressive behavior or acquired resistance to HER2-targeted therapy. These data support clinical studies of vaccination against HER3 prior to or concomitantly with other therapies to prevent outgrowth of therapy-resistant HER2+ and triple negative clones.

**Electronic supplementary material:**

The online version of this article (10.1186/s13058-018-1023-x) contains supplementary material, which is available to authorized users.

## Background

Cancer vaccines targeting well-established tumor antigens have demonstrated modest activity in clinical trials performed in the era predating effective immune checkpoint blockade. Even with more potent vaccine strategies, tumor escape may occur due to downregulation or loss of targeted antigens, as such antigens, not critical for tumor survival and proliferation, may be subject to immune editing without affecting the malignant phenotype [1]. In contrast, targeting “driver” antigens that are critical components of cellular proliferation, survival, or resistance mechanisms is an attractive strategy, as these “driver” antigens cannot be downregulated or lost due to their requirement for maintenance of the malignant phenotype. Nonetheless, the adaptive immune response against chronically overexpressed tumor antigens is often minimized or diminished due to immune tolerance and/or immunoregulation [2]. We hypothesize that a novel therapeutic strategy would be to target proteins associated with the malignant phenotype or acquired therapeutic resistance that are initially sequestered from the immune system but may become upregulated upon the initiation of therapy or tumor progression. One such upregulated mediator of therapeutic resistance is the human epidermal growth factor receptor (HER) family member HER3, associated with poor prognosis in several epithelial malignancies including breast cancer.

Although having reduced catalytic kinase activity [1–4], HER3 is thought to function as a signaling substrate for other HER proteins with which it heterodimerizes [5] thus promoting tumor proliferation and survival [6]. Importantly, it is a co-receptor for epidermal growth factor receptor (EGFR) and HER2 with which it is synergistically co-transforming [7] and rate-limiting for transformed growth [8]. Treatment of HER2-amplified breast cancers with HER2-targeting tyrosine kinase inhibitors (TKIs) promotes an increase in HER3 plasma membrane localization and downstream signaling, which can lead to resistance to the HER2-targeted therapies [9–11]. HER3 expression has been associated with poor clinical outcomes including central nervous system (CNS) metastasis in both the triple negative (TNBC) and HER2 subtypes of breast cancer [12, 13].

The pivotal role of HER3 as a hub for HER family signaling has made it an attractive therapeutic target, but its reduced kinase activity has limited the development of small molecule inhibitors. One proven method has been to disrupt the HER2-HER3 heterodimer formation. The HER2-specific monoclonal antibody pertuzumab effectively disrupts heregulin-induced HER2-HER3 dimerization and signaling [14] and has proven clinical benefit. Nonetheless, it is less effective at disrupting the elevated basal state of ligand-independent HER2-HER3 interaction and signaling in HER2-overexpressing tumor cells [15]. Alternatively, HER3 may be targeted directly, specifically with antibodies having diverse functional consequences depending on their binding site [16]. For example, HER3-specific monoclonal antibodies inhibit ligand-induced activation of the receptor [17], inhibiting downstream signaling; however, none are currently commercially available.

As an alternative to monoclonal antibodies, we and others have demonstrated that polyclonal antibodies induced by vaccination against receptors such as HER2 can recognize the cell-expressed receptor, suppress its phosphorylation, mediate profound receptor internalization and degradation, and retard the growth of established receptor-dependent tumor xenografts [18, 19]. Further, rather than repeated administration of antibodies, vaccination induces long-term anti-tumor immune responses that can be periodically boosted. Therefore, we sought to generate a vaccine capable of inducing potent anti-HER3 antibody responses.

We recently reported generation of a recombinant adenoviral vector expressing human HER3 (Ad-HER3) and demonstrated that it induced HER3-specific T cell responses and had antitumor activity [20]. However, we are aware tumor antigen-specific T cell responses may be ineffective in some patients with advanced malignancies expressing the target tumor antigen and sought evidence of alternative antitumor mechanisms. Therefore, we examined whether Ad-HER3 vaccine-induced multifunctional antibody responses, including complement-dependent cytotoxicity (CDC) and antibody-dependent cellular cytotoxicity (ADCC), may mediate antitumor responses. In addition to the expected immune-mediated functions, we also wished to demonstrate whether serum containing anti-HER3 antibody (subsequently referred to as HER3-VIA) could have a direct effect on HER3 biology, specifically mediating HER3 internalization and degradation, as well as inhibiting the downstream signaling of HER3 heterodimers. Finally, we sought to demonstrate in vivo that HER3-specific polyclonal anti-HER3 serum alone, when transferred to tumor-bearing animals, retards growth of both HER2 therapy-resistant tumors and TNBC.

## Methods

### Cell lines and cell culture

The human breast cancer cell lines BT474, MCF-7, MDA-MB-231, MDA-MB-468, SKBR3, and T47D (obtained from the American Type Culture Collection (ATCC), Manassas, VA, USA) were grown in the recommended medium. The BT474M1 human breast tumor cell line (kind gift from Dr. Mien-Chie Hung at The University of Texas M. D. Anderson Cancer Center, Houston, TX, USA) was grown in DMEM/F12 with 10% FBS. Lapatinib-resistant BT474 (rBT474) was generated as previously described [21]. Frozen stocks of these cell lines were made at earlier passages, and after thawing, cells were cultured no longer than 8 weeks for the experiments. Cells were authenticated by morphology and growth curve analysis and were routinely tested for the absence of mycoplasma by PCR. All mycoplasma tests performed during this study were negative. For tumor challenge, cell lines were tested for rodent pathogens (IMPACT Profile III) and proven to be negative before the injection to mice.

### Reagents

Trastuzumab (Herceptin™, Genentech, San Francisco, CA, USA) and cetuximab (Erbitux®, Bristol-Myers Squibb, New York, NY, USA) were purchased from the Duke University Medical Center Pharmacy. Lapatinib was purchased from Sigma-Aldrich (CDS022971, St. Louis, MO, USA). Heregulin (377-HB/CF), heregulin with a C-terminal 6-His tag (5898-NR) and allophycocyanin (APC)-conjugated anti-His Tag antibody (IC050A) were purchased from R&D Systems (Minneapolis, MN, USA).

### Adenovirus vector preparation

The human HER3 complementary DNA (cDNA) was excised from a pCMVSport6-HER3-HsIMAGE6147464 plasmid (cDNA clone MGC:88033/IMAGE:6147464 obtained from ATCC). Construction of a first-generation (E1-, E3-) Ad vector containing human full length HER3 under control of human cytomegalovirus (CMV) promoter/enhancer elements was performed using the pAdEasy system (Agilent technologies, Santa Clara, CA, USA) as previously described [22–24]. Similar Ad-vectors containing the green fluorescence protein (GFP) or lacZ rather than HER3 was similarly generated to serve as controls.

### Mice

BALB/c and NOD.CB17-*Prkdc*^*scid*^/J mice were purchased from Jackson Labs (Bar Harbor, ME, USA). All mice were maintained under specific pathogen-free conditions, and all work was conducted in accordance with Duke Institutional Animal Care and Use Committee (IACUC)-approved protocols.

### Production of vaccine-induced antibodies (VIA)

BALB/c mice were vaccinated on day 0 and day 14 by footpad injection of Ad-GFP (control), or Ad-HER3 vectors (2.6 × 10^10^ particles/ mouse). At 14 days after the second vaccination, mice were euthanized and serum was collected, pooled from every 20 vaccinated mice, and 1-mL aliquots were made and stored at − 80 °C until use. Approximately 80 mice for HER3-VIA and 80 mice for control VIA were vaccinated to collect and pool serum (24 mL for both VIAs) for this study.

### Cell-based ELISA

The 4 T1 cells were transduced with the HER3 gene by lentiviral vectors (4 T1-HER3 cell): 4 T1 and 4 T1-HER3 cells were incubated overnight at 37 °C in 96-well plates (3 × 10^4^ cells/well). Mouse serum (HER3-VIA, LacZ-VIA, GFP-VIA) was diluted (final titrations 1:50 ~ 1:6400), added to the wells (50 μL/well), and incubated for 1 h on ice. The plates were washed with PBS twice, and then cells were fixed with diluted formalin (1:10 dilution).Then, near infrared (nIR) dye-conjugated anti-mouse IgG (IRDye 800CW, LI-COR Biosciences, Lincoln, NE, USA) was added (1:2000 dilution, 30 min, room temperature). After washing with PBS, the nIR signal was detected by a LI-COR Odyssey Imager (LI-COR) at 800 nm channel.

### Analysis of anti-HER3 antibody binding by flow cytometry

HER3 vaccine-induced antibodies in vaccinated mouse serum were measured by flow cytometry as reported [25]. Briefly, 3 × 10^5^ human breast cancer cells were incubated with diluted (1:100 to 1:51200) mouse, post-vaccine serum (HER3-VIA or GFP-VIA) for 1 h at 4 °C and then washed with 1% BSA-PBS. The cells were further stained with phycoerythrin (PE)-conjugated anti-mouse IgG (Dako, catalog number R0480) for 30 min at 4 °C and washed again. Samples were analyzed on a BD LSRII flow cytometer (Becton Dickenson, San Jose, CA, USA) and mean fluorescence intensity (MFI) reported. For the analysis of HER family expression on tumor cells, PE-conjugated anti-EGFR, anti-HER2 (BD Biosciences), and anti-HER3 antibody (BioLegend) were used as in the manufacturer’s instructions.

### Detection of HER3 epitopes bound by vaccine-induced antibody

Epitopes were mapped using spotted peptide arrays of 15-mer peptides overlapping by four amino acids representing the full length of the human HER3 protein. HER3 peptides were coated onto cellulose membranes using a Spot Robot ASP 222 (AbiMed) and HER3-VIA (1:100 dilution in saline) epitopes were mapped as described [26].

### Heregulin binding assay

BT474 cells (HER3+) were incubated with medium containing no serum at 37 °C for 24 h. At 30 min before the assay, the culture plates were placed at 4 °C to avoid internalization of HER3 receptor. The following procedures were performed on ice. Cells were pre-incubated with heregulin (final concentrations: 0, 10, 100 nM) or GFP-VIA/HER3-VIA (final dilution: 1:100) for 10 min, then heregulin-His Tag (final concentration: 100 nM) was added and further incubated for 10 min. After washing three times with cold PBS, cells were incubated with APC-conjugated anti-His Tag antibody for 30 min. Cells were washed with cold PBS three times, harvested from the flask, and analyzed using the LSRII flow cytometer.

### MTT assay to detect cell proliferation

The effect of HER3-VIA on the proliferation of human breast cancer cell lines was measured as previously described [27]. Briefly, 5000 cells per well were cultured in a 96-well plate with HER3-VIA (1:33 dilution), GFP-VIA (1:33 dilution) or trastuzumab 20 μg/ml for 3 days and proliferation was assessed by a 3-(4,5-dimethylthiazol-2-yl)-2.5-diphenyltetrazolium bromide (MTT) assay.

### Assessment of HER3 internalization

Human HER3+ breast cancer cells (SKBR3, BT474M1 and MDA-MB-468) were incubated with 1:100 HER3-VIA or GFP-VIA at 37 °C for 60 min (SKBR3, BT474M1) or 3 h (MDA-MB-468). After washing, fixation with 4% paraformaldehyde (PFA), and application of permeabilizing solution 2 (Becton Dickenson), nonspecific binding was blocked with 2.5% goat serum at 37 °C for 30 min. Cells were incubated with 1:100 Red™-conjugated anti-mouse IgG (H + L) (Jackson ImmunoResearch Laboratories Inc. West Grove, PA, USA) in a dark chamber for 1 h at room temperature and washed with PBS. For the detection of EGFR, MDA-MB-468 cells were incubated with HER3-VIA for 3 h, then fixed and permeabilized. Cells were then labeled with cetuximab (40 μg/mL) for 10 min, followed by staining with Cy2-conjugated anti-human IgG antibody (ab97169, Abcam, Cambridge, MA, USA). For the detection of HER2, SKBR3 cells were labeled with trastuzumab (40 μg/mL) for 10 min, washed with medium, and then incubated with HER3-VIA for 3 h. Then, cells were fixed and permeabilized, and stained with Cy2-conjugated anti-human IgG antibody. Slides were mounted in VectaShield containing 4′,6-diamidino-2-phenylindole (DAPI) (Vector Laboratories, Burlingame, CA, USA) and images were acquired using a Zeiss Axio Observer wide-field fluorescence microscope (Carl Zeiss, München-Hallbergmoos, Germany).

### Complement-dependent cytotoxicity assay

We performed complement-dependent cytotoxicity assays using our previously published protocol [27]. Briefly, target cells were incubated with rabbit serum (1:100) as a source of complement and the HER3-VIA or GFP-VIA in serum from mice immunized as above diluted (1:100), or trastuzumab (20 μg/mL) at 37 °C for 2 h. After incubation, cytotoxicity was measured using the CytoTox 96 Nonradioactive Cytotoxicity Assay (Promega; per manufacturer’s instructions) to measure lactate dehyrdrogenase (LDH) release in the culture medim as evidence of cytotoxicity.

### Antibody-dependent cell-mediated cytotoxicity assay

Antibody-dependent cell-mediated cytotoxicity was measured against HER3-expressing JC-HER3 cells and parental JC cells (HER3-negative) using mFcγRIV ADCC Reporter Bioassay (Promega, catalog number M1211). Target cells (25,000 or 10,000 cells/well) were seeded into 96-well plates, incubated overnight, and pre-incubated with 1:10 dilution of HER3-VIA or GFP-VIA for 30 min at room temperature. Then, effector cells were applied per manufacturer’s instructions. Two different effector-target ratios (3:1, 7.5:1) were tested. After 6 h of co-incubation, Bio-Glo™ Reagent was added, and luminescence was measured. Luminescence of JC cells was subtracted from luminescence of JC-HER3 cells for each VIA.

### Treatment of established HER3+ human breast tumor xenografts by passive transfer of vaccine-induced antibodies

BT474M1 cells, lapatinib-resistant rBT474 cells, or MDA-MB-468 cells (5 × 10^6^, 1 × 10^6^, 1 × 10^6^ cells/mouse, respectively) were implanted in the mammary fat pads of 8–10-week-old NOD.CB17-*Prkdc*^*scid*^/J mice. At 2 days prior to tumor implantation, 17-beta-estradiol pellets (0.72 mg 60-day continuous release pellets; Innovative Research of American, Sarasota, FL, USA) were subcutaneously implanted in the backs of the mice, except for the experiment with MDA-MB-468 cells. Tumors were allowed to develop for 14 days (BT474M1), 2 months (rBT474), or 12 days (MDA-MB-468), to reach the volume of approximately 50–100 mm^3^, and then mice were randomized to receive intravenous injection of either GFP-VIA or HER3-VIA. VIA (100–150 μL) was injected at 2–3-day intervals for a total of 10 administrations. Tumor growth was measured in two dimensions using calipers and tumor volume was determined using the formula:

$$ \mathrm{Volume}=\frac{1}{2}\ \left[{\left(\mathrm{Width}\right)}^2\mathrm{x}\ \left(\mathrm{Length}\right)\right]. $$

### Western blotting to analyze pathway inhibition

Tumors were isolated from euthanized, VIA-treated mice and immediately flash frozen. Tissue extracts were prepared as previously described [27]. Equal amounts of proteins (50 μg) were resolved by 4–15% gradient SDS PAGE. After transfer, membranes were probed with specific antibodies recognizing target proteins: pTyr (Sigma), ErbB2, ErbB3, Akt, pAkt473, Erk 1/2, pErk1/2 (Cell Signaling, Beverly, MA, USA), survivin, actin (Sigma, St. Louis, MO, USA), 4EBP-1, p4EBP-1, s6, ps6 (Santa Cruz Biotech, Santa Cruz, CA, USA), and then with IRDye 800 conjugated anti-rabbit or mouse IgG or Alexa Fluor 680 anti-rabbit IgG, and were visualized using the Odyssey Infrared Imaging System (LI-COR, Lincoln, NE, USA) as previously described [27].

### Immunohistochemical analysis of HER3 expression in tumor tissue

BT474M1 or rBT474 tumors were collected when mice were sacrificed, fixed with 10% neutral-buffered formalin, and embedded to paraffin. Tissue sections of 4 μm thick were deparaffinized, and heat-induced antigen retrieval was performed in sodium citrate buffer for 20 min. After blocking endogenous peroxidase activity with 3% H_2_O_2_, 10% normal horse serum was applied for the blocking of nonspecific binding sites. Anti-HER3 antibody (Santa Cruz) was put on the sections and incubated overnight at 4 °C. After washing with PBS, biotinylated secondary antibody (Bio-Rad) was applied for 30 min, followed by an VECTASTAIN ABC kit (Vector Lab) and then the color was developed using the DAB Peroxidase substrate kit (Vector Lab). Counterstaining was performed with hematoxylin.

### Statistical analysis

Tumor volume over time was standardized by the baseline tumor volume. Area under the tumor growth curve was calculated under spline interpolation [28] and adaptive quadrature. Groups were compared based on the Kruskal-Wallis test [29] followed by multiple comparisons performed by the non-parametric Tukey test [30]. If only two groups were compared then the Mann-Whitney test [31] was applied. Normality assumption was verified by the Shapiro-Francia test [32] and homogeneity of variances by the Levene test [33]. Difference in luminescence intensity in the ADCC assay was analyzed by Fisher’s exact test. All tests of hypotheses were two-sided, with a significance level of 0.05. Calculations were performed using R, version 3.2.5 [34].

## Results

### Ad-HER3 activates anti-HER3 antibody responses

**Fig. 1:**
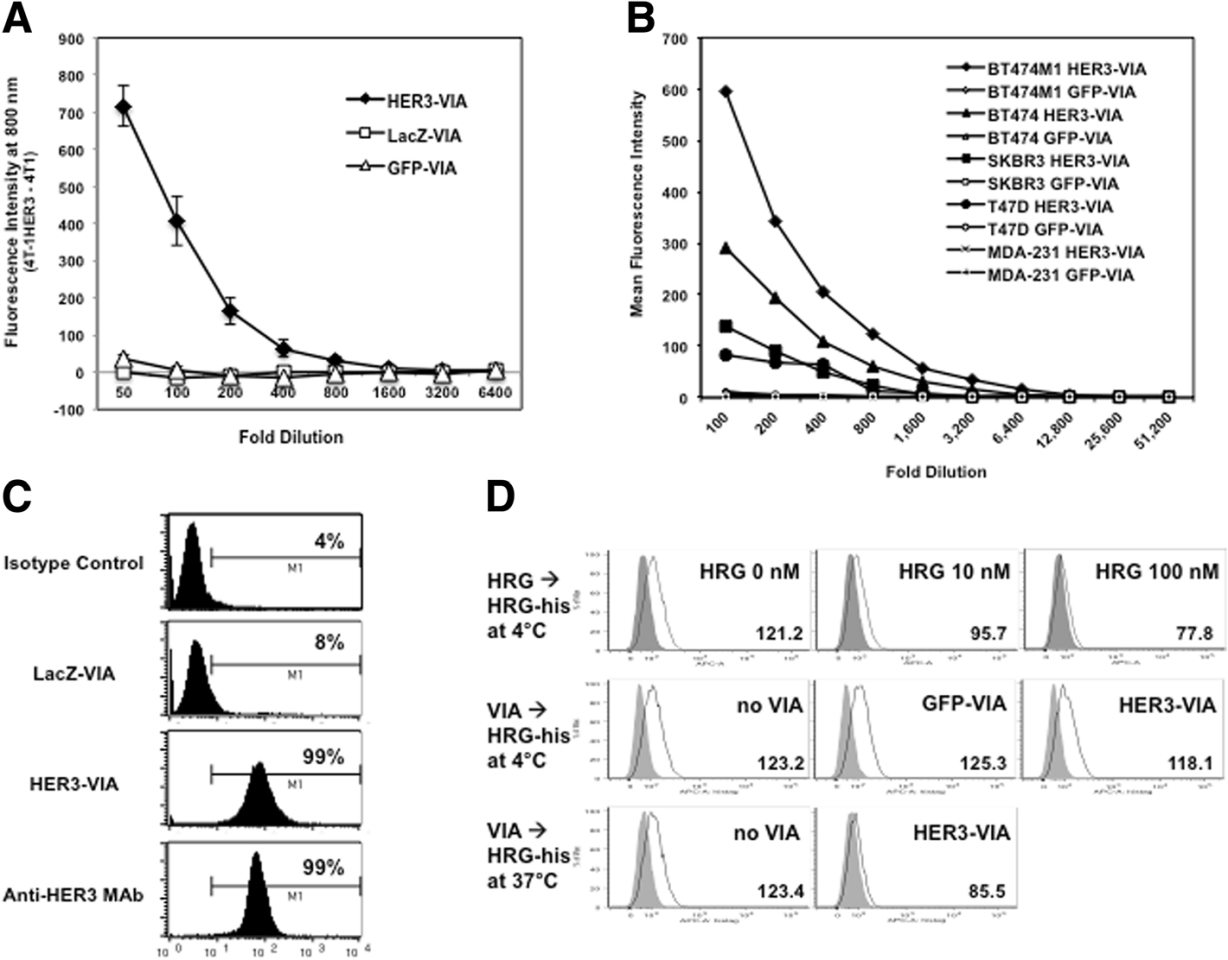
Human epidermal growth factor receptor 3 **(**HER3)-specific antibody responses are induced by adenovirus encoding full length human HER3 (Ad-HER3) in vivo. **a** Binding of vaccine-induced antibody (VIA) in serum from mice immunized with Ad-HER3 (HER3-VIA), Ad-lacZ (lacZ-VIA), and Ad-green fluorescence protein (GFP-VIA). Serum at dilutions presented were mixed with the HER3-4 T1 cell line or wild-type 4 T1 and binding of antibody was identified with near infrared (nIR) dye-conjugated anti-mouse IgG and detected by a LI-COR Odyssey Imager. The difference in fluorescence intensity between HER3-4 T1 and 4 T1 is graphed. **b** Binding of HER3-VIA to breast cancer cell lines: a panel of human breast cancer cell lines were incubated with dilutions of the HER3-VIA, washed, and then mixed with a phycoerythrin (PE)-conjugated secondary antibody. HER3-VIA binding was analyzed based on fluorescence activated cell sorting analysis and mean fluorescence intensity was reported. **c** Flow cytometric analysis was used to identify percentage of BT474 cells able to bind HER3-VIA (1:100 dilution). **d** Binding of His-tagged heregulin to HER3 receptor was measured by pre-incubating BT474 cells with heregulin (0, 10, 100 nM), or HER3-VIA (1:100) for 10 min on ice, followed by incubation with His-tagged heregulin (100 nM) for 10 min. Receptor-bound His-tagged heregulin was visualized by staining with PE-conjugated anti-His Tag antibody. Mean fluorescence intensity is shown in each histogram

We first assessed the antibody response to the Ad-HER3 vaccine. We vaccinated BALB/c mice twice at a 14-day interval with a recombinant E1-, E3- adenovirus serotype 5 vector expressing full length human HER3 (Ad-HER3) and 14 days later collected the serum for analysis. To detect HER3-specific antibodies that could recognize membrane-associated HER3, binding of vaccine-induced antibodies (VIA) in mouse serum was first tested using a cell-based ELISA against a murine triple negative breast cancer cell line (4 T1) transduced to express HER3 (4 T1-HER3) (Fig. 1a**).** Mice vaccinated with Ad-HER3 had serum titers > 1:800 against the 4 T1-HER3 cells, in contrast to the mice receiving control Ad-LacZ and Ad-GFP vaccines, which only displayed background levels of binding (Fig. 1a**)**. We then tested HER3-VIA for binding to a series of human HER3-expressing breast tumor cell lines, including the high HER3-expressing BT474M1 and BT474, the moderately HER3-expressing SKBR3 and T47D, and the HER3 low/negative, triple negative MDA-MB-231 tumor cell lines (Fig. 1b). Serum from Ad-HER3-vaccinated mice demonstrated titers > 1/800 against the human high HER3-expressing cell lines and there was no binding to the HER3 low/negative cell lines (Fig. 1b). The apparent titer correlated with the extent of HER3 expression in the cell lines (Additional file 1: Figure S1).

To confirm the binding of the HER3 vaccine-induced antibodies (HER3-VIA), we also performed flow cytometric analysis of HER3-expressing BT474 exposed to the HER3-VIA serum followed by a secondary antibody to detect the presence of HER3 bound antibody (Fig. 1c): 99% of the BT474 cells stained positively with the HER3-VIA, similar to that seen with a commercially available monoclonal antibody. These data demonstrate that antibodies within the HER3-VIA bind to endogenous HER3-expressed on human breast cancer cell lines.

To demonstrate that the HER3-VIA response was polyclonal, and to confirm that multiple HER3 epitopes could be recognized, we tested pooled HER3-VIA for binding to a series of HER3 peptides displayed as peptide arrays. The HER3-VIA recognized at least 18 epitopes in both the intracellular and extracellular domain of HER3 (Additional file 2: Table S1). Surprisingly none of these epitopes included the reported heregulin binding site [35]. It should be noted that peptide arrays do not recapitulate conformationally correct protein structures. Therefore, we wished to confirm that HER3-VIA did not block the binding of the ligand, heregulin, to HER3. To detect the binding of heregulin we used His-tagged heregulin, which could be detected by fluorescently conjugated anti-His Tag antibody. We confirmed that the His-tagged heregulin could occupy the heregulin binding site on HER3 by demonstrating that it could compete with untagged heregulin in a concentration-dependent fashion in BT474 cells (Fig. 1d). His-tagged heregulin binding to HER3 on BT474 cells was not affected by HER3-VIA when the assay was performed on ice to maintain the receptor on the cell surface (Fig. 1d). Taken together, these data demonstrate that the polyclonal HER3-VIA binds to multiple sites on HER3, but not the heregulin binding site.

### HER3-VIA mediates complement-dependent cytotoxicity

**Fig. 2:**
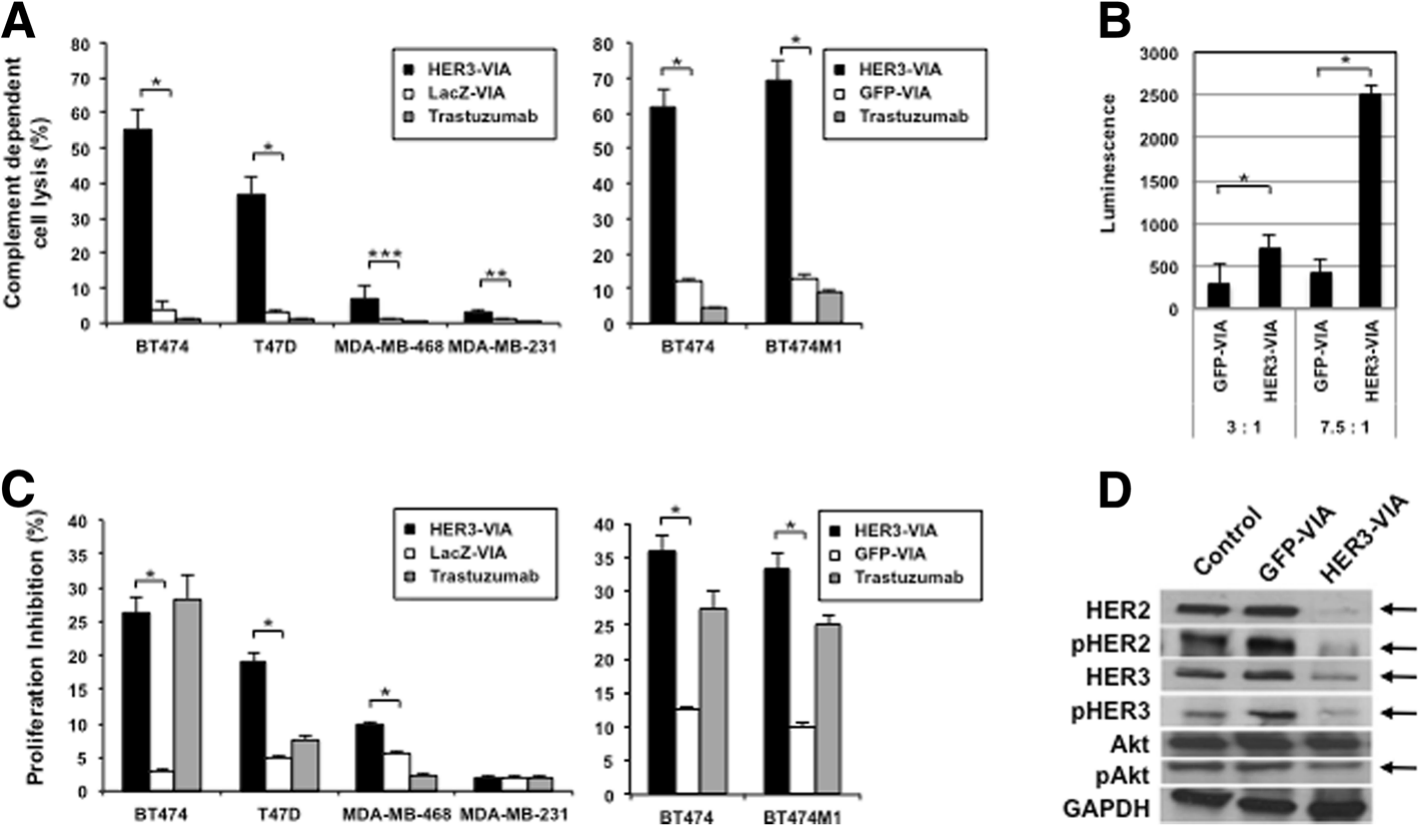
Vaccine-induced antibody (VIA) in serum from mice immunized with adenovirus encoding full length human epidermal growth factor receptor 3 (HER3-VIA) mediates multiple mechanisms of action against human breast tumor cell lines in vitro. **a** Complement dependent cytotoxicity was measured against human epidermal growth factor receptor 3 (HER3)-expressing (BT474, T47D, MDA-MB-468, BT474M1) and HER3-negative (MDA-MB-231) cell lines. Percentage cytolysis is reported: *p < 0.0001, **p < 0.001, ***p < 0.05. **b** Antibody-dependent cell-mediated cytotoxicity was measured against HER3-expressing JC-HER3 cells and parental JC cells (HER3-negative) using mFcγRIV antibody-dependent cellular cytotoxicity (ADCC) Reporter Bioassay. Target cells were pre-incubated with 1:10 dilution of HER3-VIA or green fluorescence protein (GFP)-VIA for 30 min at room temperature, and then effector cells were applied per manufacturer’s instructions. Two different effector-target ratios (3:1, 7.5:1) were tested. After 6 h of co-incubation, Bio-Glo™ Reagent was added, and luminescence was measured. Luminescence of JC cells was subtracted from luminescence of JC-HER3 cells for each VIA and is shown: *p < 0.0001. **c** Proliferation of HER3-expressing (BT474, T47D, MDA-MB-468, BT474M1) or HER3-negative cell line (MDA-MB-231) was measured in a 72-h 3-(4,5-dimethylthiazol-2-yl)-2.5-diphenyltetrazolium bromide (MTT) assay in response to HER3-VIA or control (LacZ-VIA or GFP-VIA): *p < 0.0001. **d** Effect of HER3-VIA on HER3 expression and signaling pathway was analyzed by western blotting. BT474M1 cells were incubated with HER3-VIA or GFP-VIA (1:100 dilution) in vitro at 37 °C for 3 h. GAPDH, glyceraldehyde-3-phosphate dehydrogenase

Having demonstrated that the HER3-VIA contains antibodies with multiple specificities, we were interested to determine whether this resulted in multiple different functions. Because direct antibody-mediated tumor cell binding and killing is an established mechanism of action of antibodies induced by vaccination, we evaluated the capacity of HER3-VIA to mediate complement-dependent cytotoxicity (CDC). HER3-VIA exhibited significant CDC against a number of HER3-expressing human breast tumor cells but not the HER3-negative MDA-MB-231 cell line, while control LacZ-VIA did not mediate CDC (Fig. 2a). There was lower but detectable CDC against the HER3+ triple negative cell line MDA-MB-468. Further, HER3-VIA mediated similar levels of CDC against BT474 and its metastatic variant BT474-M1. As previously observed, trastuzumab did not mediate CDC. These data demonstrate immune-mediated anti-tumor activity for HER3-VIA.

### HER3-VIA induces antibody-dependent cell-mediated cytotoxicity (ADCC)

We tested the ability of Ad-HER3 VIA to mediate ADCC against HER3-expressing tumor cells. ADCC activity was measured through the detection of mFcγRIV-mediated signaling in a reporter bioassay. With a 1:10 dilution of HER3-VIA and GFP-VIA, there was significantly stronger activation of the mFcγRIV-mediated signaling pathway when HER3-VIA was co-incubated with effector and target cells, suggesting that HER3-VIA can mediate killing of HER3-expressing cells through ADCC (Fig. 2b).

### Anti-proliferative effects of HER3-VIA in vitro

Although immunization with Ad-HER3 did induce antibodies with expected immune function (CDC and ADCC), we also wished to determine whether the induced antibodies could inhibit tumor cell proliferation through effects on signaling pathways governed by HER3. We found that when human breast cancer cells highly expressing HER3 (such as BT474 and highly metastatic variant BT474M1) were cultured with HER3-VIA, their proliferation was significantly inhibited compared with cells cultured with control LacZ-VIA (Fig. 2c). Of interest, the HER2/HER3 expressing tumor cells were similarly responsive to the growth inhibitory effect of the HER3-VIA as they were to the anti-HER2 antibody trastuzumab. The MDA-MB-468 cells moderately expressing HER3 and the HER3-negative MDA-MB-231 cells had only slight (~ 10%) and no growth inhibition by HER3-VIA, respectively, suggesting that the growth inhibitory effect of the HER3-VIA was correlated with HER3 expression. These data demonstrate that the HER3-VIA has anti-tumor functionality beyond the expected immune-mediated cell killing.

### Downregulation of HER3 expression and signaling inhibition by HER3-VIA

**Fig. 3:**
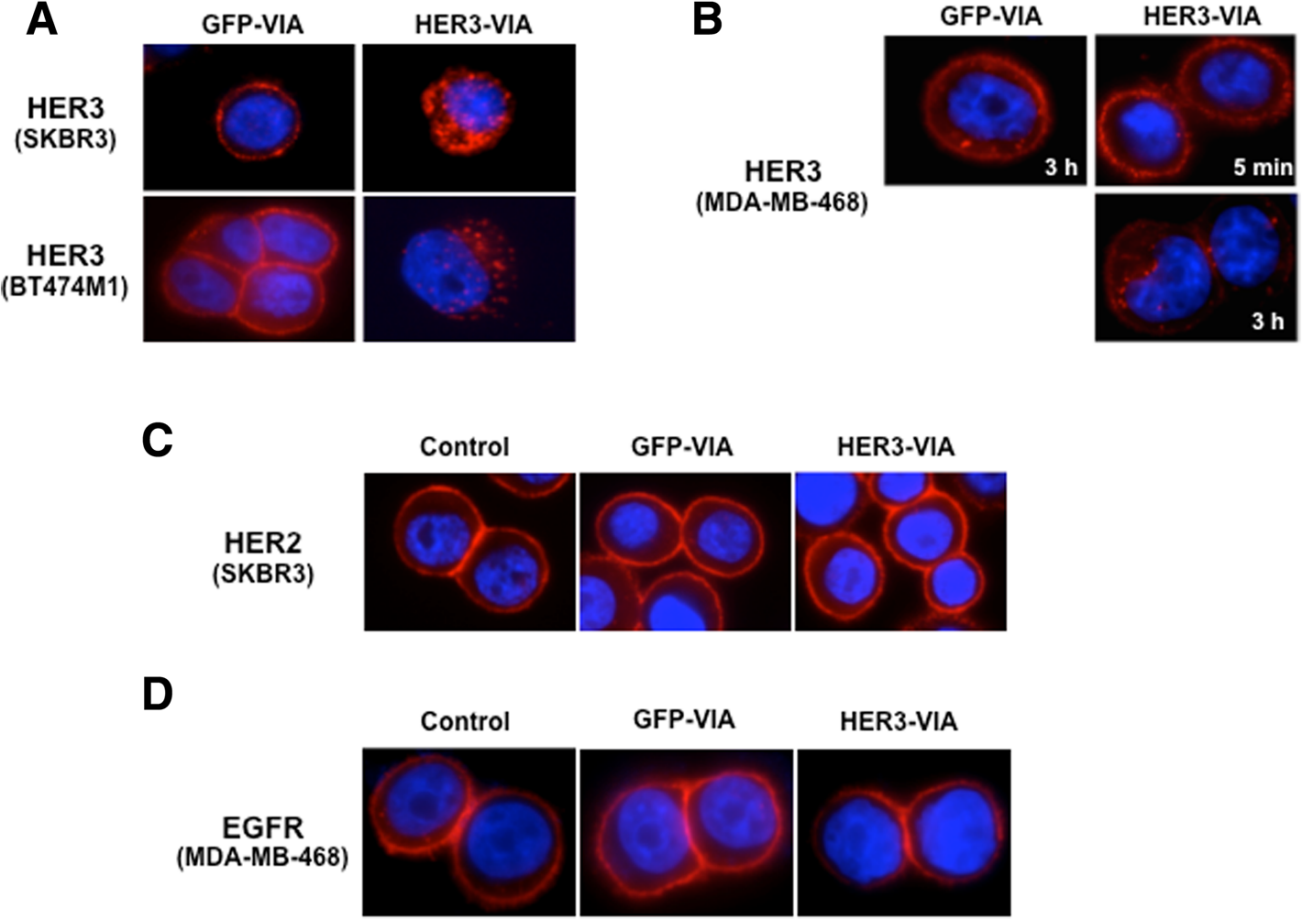
Vaccine-induced antibody (VIA) in serum from mice immunized with adenovirus encoding full length human 3 epidermal growth factor receptor (HER3-VIA) induces receptor internalization and downregulation of human epidermal growth factor receptor 3 (HER3), but not epidermal growth factor receptor (EGFR) nor HER2. SKBR3, BT474-M1 (**a**) and MDA-MB-468 (**b**) were incubated with 1:100 HER3-VIA or green fluorescence protein (GFP)-VIA at 37 °C for 1 or 3 h, respectively. After washing and permeabilization, RedTM-conjugated anti-mouse IgG (H + L) was used to visualize internalization of the VIA-bound proteins with a fluorescence microscope. SKBR3 cells (**c**) or MDA-MB-468 cells (**d**) were incubated with 1:100 HER3-VIA or GFP-VIA at 37 °C for 3 h. Cells were fixed, permeabilized and stained with anti-HER2 antibody (trastuzumab, 40 μg/mL) (**c**) or anti-EGFR antibody (cetuximab, 40 μg/mL) (**d**), followed by Cy2-conjugated anti-human IgG antibody

Growth factor receptor downregulation by internalization and degradation has been proposed as a mechanism for the inhibition of tumor growth mediated by monoclonal antibodies. BT474M1 cells exposed in vitro to HER3-VIA demonstrated downregulation of HER3 and HER2 expression and inhibition of pHER3 and pAkt signaling (Fig. 2d). To ascertain whether receptor downregulation was caused by HER3-VIA and was a consequence of receptor internalization, we visualized cell membrane-associated HER3 on SKBR3 and BT474M1 tumor cells. When exposed to serum containing either HER3-VIA or GFP-VIA, the HER3-VIA exposure resulted in dramatic internalization and aggregation of the receptor within 1 h after exposure to HER3-VIA, but this was not observed with exposure to control GFP-VIA (Fig. 3a). Similar results were observed for the HER3-expressing, TNBC cell line MDA-MB-468 when exposed to the HER3-VIA (Fig. 3b). These data demonstrate that the HER3-activated polyclonal antibodies are able to mediate HER3 internalization in both HER2-positive and TNBC cells.

There are two consequences of the downregulation of HER3; one is the lack of sites for ligand binding and the other is the loss of a heterodimer partner for EGFR or HER2. Indeed, we observed that when BT474 cells were pre-incubated with HER3-VIA for 3 h at 37 °C, the amount of bound His-tagged heregulin was much smaller, suggesting that HER3 was downregulated by VIA-induced internalization (Fig. 1d). These data suggest that the mechanism of action for the anti-proliferative effect of HER3-VIA is not interference with ligand binding but rather cell surface receptor loss interfering with signaling.

Receptor tyrosine kinases of the EGFR family are known to form homodimers or heterodimers. Therefore, we tested the effect of HER3-VIA on HER2 and EGFR expression using fluorescence microscopy (Fig. 3c, c). For some cancer cell lines, such as BT474M1, HER2 was slightly downregulated after 3 h incubation with HER3-VIA (Fig. 2d), but the trend was not clear in other cell lines. The inconsistency may derive from the different environments, such as production of heregulin by cancer cells, because heregulin will induce the heterodimerization of HER2/HER3. For SKBR3 cells, HER2 internalization was not evident (Fig. 3c). We did not find any significant effect for the EGFR expression on cell surface by exposure to HER3-VIA (Fig. 3d). Other monoclonal antibodies (trastuzumab, cetuximab) and small molecule inhibitor (lapatinib) as well as GFP-VIA/HER3-VIA were examined for their effect on cell surface expression of EGFR, HER2 and HER3 by flow cytometry (Additional file 3: Figure S2). Trastuzumab and Cetuximab did not decrease the surface expression of their target HER receptors, while HER3-VIA substantially decreased the surface expression of HER3. Lapatinib induced slight decrease of EGFR expression, and gradually enhanced HER2 and HER3 expression on the cell surface by the 24 h time point. Thus, the downregulation of cell surface HER3 was specific with HER3-VIA treatment.

### Inhibition of HER2+/HER3+ tumor growth by HER3-VIA in vivo

**Fig. 4:**
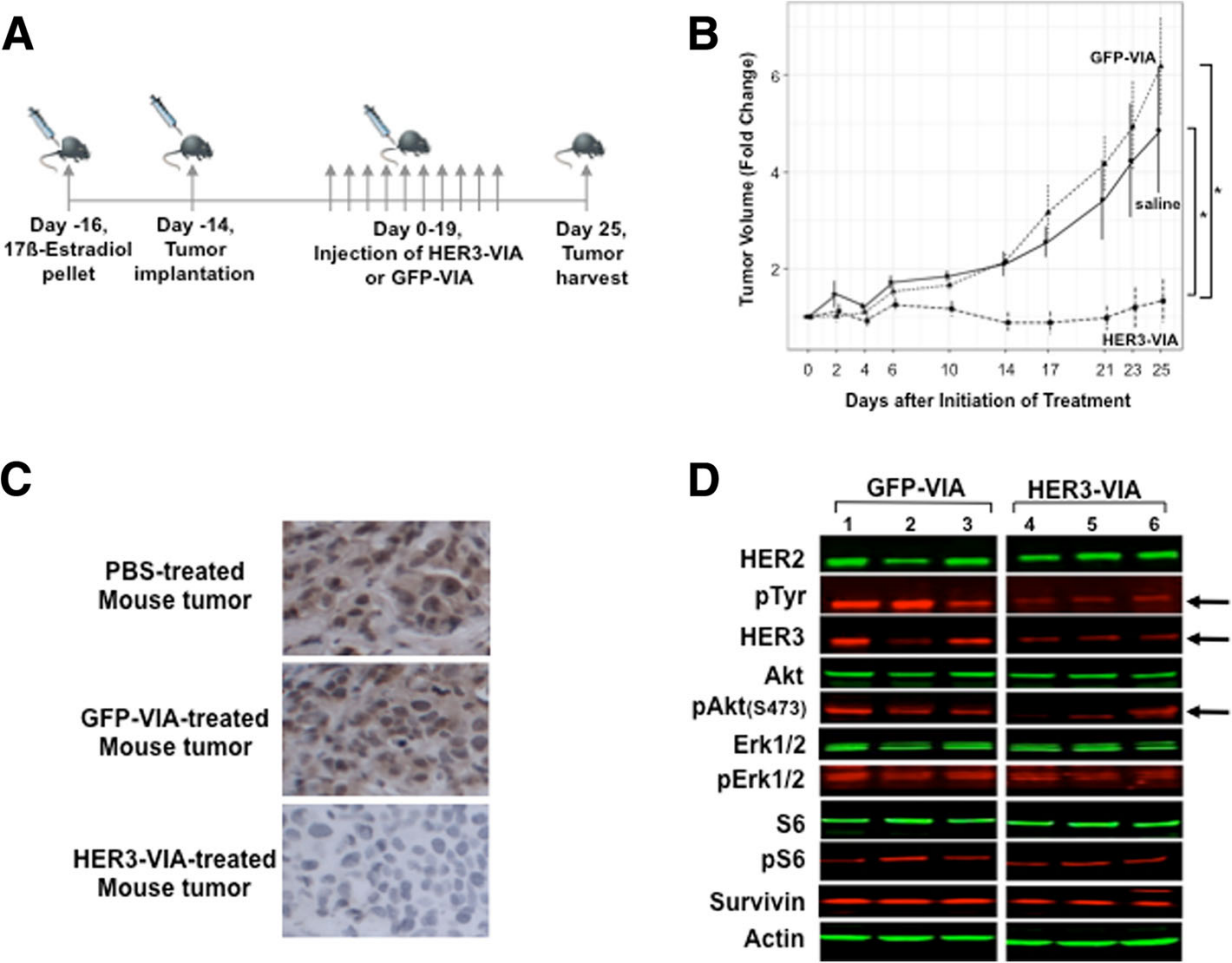
In vivo effects of human epidermal growth factor receptor 3 (HER3) vaccine-induced antibody (HER3-VIA) on BT474M1 human breast tumor xenografts. **a** The experiment schema is shown. Tumor cells were implanted into SCID mice on day 0 and then HER3-VIA or control green fluorescence protein (GFP)-VIA was transferred via tail vein injection on day 14–33. **b** HER3-VIA retarded the growth of established BT474M1 breast cancers: *p < 0.001). **c** Immunohistochemical analysis of HER3 protein expression in excised tumors following HER3-VIA administration. **d** Western blot analysis of excised tumors probing for downstream signaling intermediaries of HER3 activation

After finding that HER3-specific antibodies inhibited HER3+ tumor cell proliferation in vitro, we sought to demonstrate the effects of HER3-VIA in vivo against the metastatic human xenograft model BT474M1 that expresses both HER2 and HER3 (Fig. 4a**)**. The mouse model that allowed engraftment of human tumor cells lack functional T and B cells, but also lack complement and ADCC effector cells, so these studies should focus on the direct antitumor functions of HER3 VIA. Systemically administered HER3-VIA prevented the growth of the established BT474M1 (*p* < 0.001) when compared to the control GFP-VIA-treated mice (Fig. 4b). Further, residual tumor in mice treated with HER3-VIA had decreased levels of HER3, indicating either downregulation of HER3 or elimination of tumor cells expressing HER3 (Fig. 4c). The decrease in HER3 expression following HER3-VIA administration was associated with a decrease in pHER2 (pTyr) and pAKT (Fig. 4d). These data demonstrate that the polyfunctional anti-HER3 antibodies downregulate HER3 expression and signaling in tumors overexpressing HER2/HER3.

### Inhibition of therapy-resistant tumor growth by HER3-VIA in vivo

**Fig. 5:**
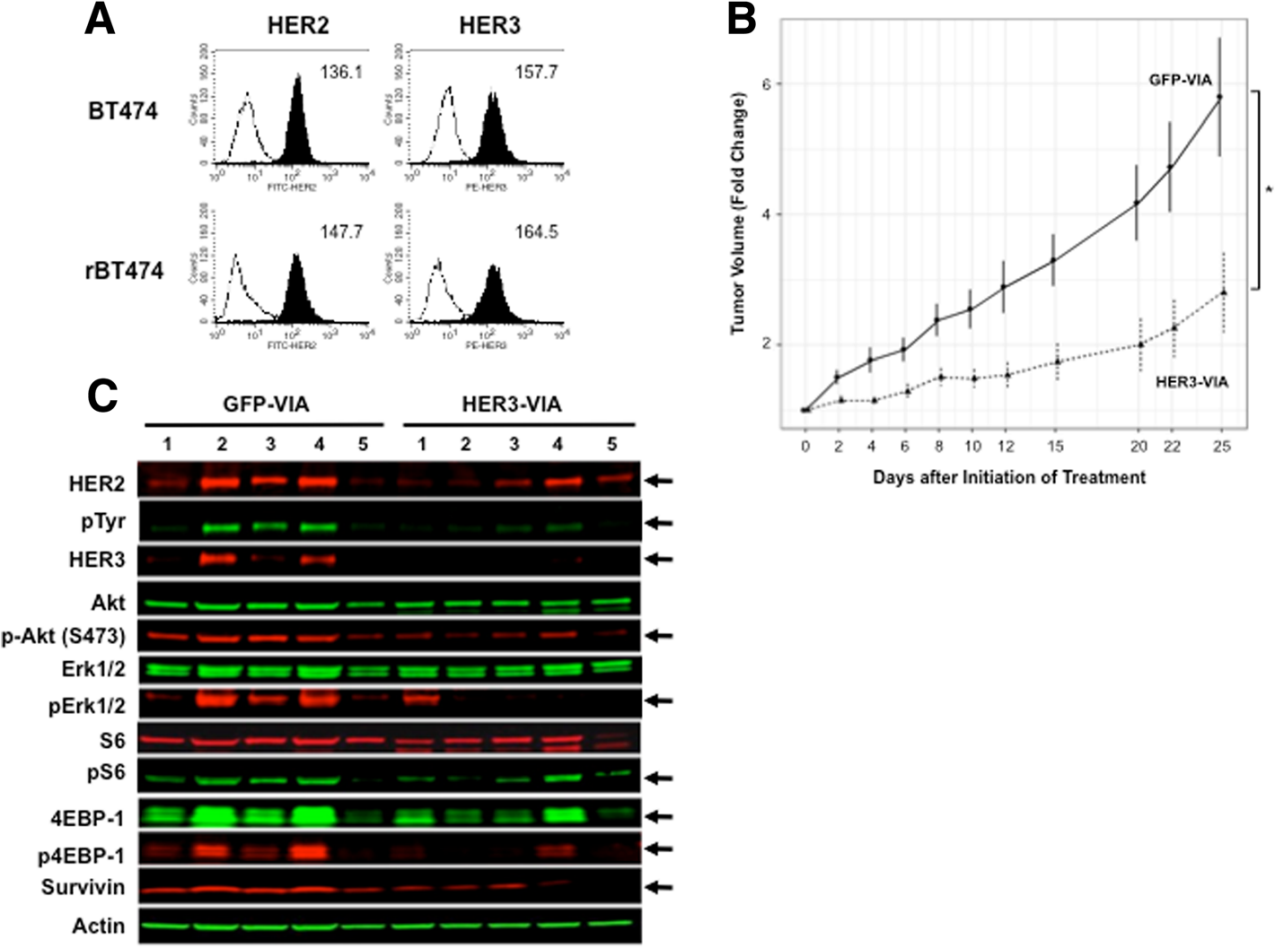
In vivo effects of human epidermal growth factor receptor 3 (HER3) vaccine-induced antibody (HER3-VIA) in lapatinib-refractory rBT474 SCID tumor xenografts. **a** Flow cytometry analysis of HER2/HER3 expression (filled histograms) by BT474 and rBT474 cells. Mean fluorescence intensities are shown in each histogram. **b** The lapatinib-refractory cell line rBT474 was implanted into SCID mice and HER3-VIA was administered and tumor size was measured every 2-3 days : *p < 0.01). **c** Western blot analysis of excised tumors probing for downstream signaling intermediaries of HER3 activation. GFP, green fluorescence protein

Having demonstrated anti-tumor activity for HER3-VIA in parental breast cancer cells lines, we extended our work to evaluate whether anti-tumor activity could be demonstrated against resistant variants, specifically tumor cells resistant to HER2-targeting therapies. Therefore, we tested the effects of HER3-VIA using a lapatinib-resistant cell line (rBT474), which we confirmed expresses HER2 and HER3 at similar levels to the BT474 tumor line (Fig. 5a), derived as previously reported [20]. The HER3-VIA retarded the growth of the established lapatinib-resistant tumors (*p* < 0.01) (Fig. 5b). As observed for the parental cell line, the residual laptinib-resistant tumor had decreased expression of HER3 (Fig. 5c). Decreased HER2:HER3 signaling following HER3-VIA was demonstrated by the deceased pTyr, pAkt473(S473), pErk1/2, p4EBP1, survivin and pS6, relative to the control GFP-VIA-treated tumors. These data demonstrate that the HER3-VIA antibody response to Ad-HER3 immunization has therapeutic potential against treatment-resistant cell lines.

### Inhibition of TNBC growth by HER3-VIA in vivo

**Fig. 6:**
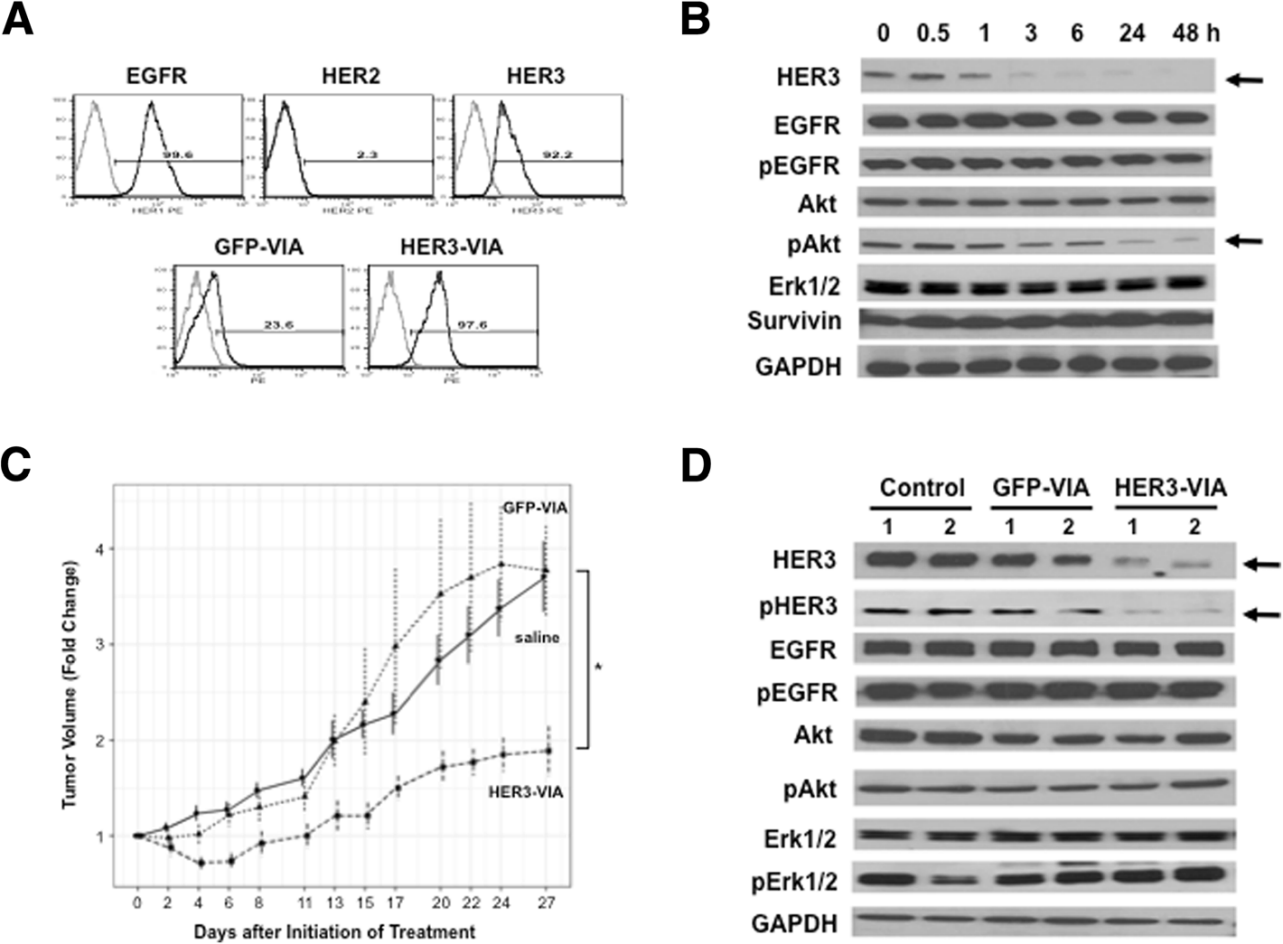
In vivo effects of human epidermal growth factor receptor 3 (HER3) vaccine-induced antibody (HER3-VIA) in triple negative MDA-MB-468 SCID tumor xenografts. **a** Flow cytometry analysis of HER family expression by MDA-MB-468 cells. Cells were stained with phycoerythrin (PE)-conjugated anti-epidermal growth factor receptor (anti-EGFR), anti-HER2, or anti-HER3 monoclonal antibodies (mAb) (upper three histograms), or were incubated with HER3-VIA or green fluorescence protein (GFP)-VIA (1:100 dilution), followed by PE-conjugated anti-mouse IgG (lower two histograms). **b** Effect of HER3-VIA on signaling pathway was analyzed by western blotting. MDA-MB-468 cells were incubated with HER3-VIA (1:100 dilution) in vitro at 37 °C for the indicated time period. **c** Passive transfer of HER3-VIA retarded the growth of established MDA-MB-4684 in SCID mice: *p < 0.001). **d** Western blot analysis of the in vivo signaling effects of HER3-VIA exposure compared to control and GFP-VIA in triple negative MDA-MB-4684 SCID tumor xenografts. GAPDH, glyceraldehyde-3-phosphate dehydrogenase

Because a subset of TNBC express HER3, we wished to determine if the antitumor activity observed for HER3-VIA in HER2-expressing cell lines would also be observed in TNBC cell lines. EGFR is known to heterodimerize with HER3 on triple negative MDA-MB-468 cells. We confirmed the expression of EGFR and HER3 on MDA-MB-468 cells, and binding of HER3-VIA to the cells by flow cytometry (Fig. 6a). We also tested the effect of HER3-VIA treatment on the signaling pathway in MDA-MB-468 cells in vitro. In this study, cells were incubated with HER3-VIA (1:100 dilution in the culture medium) at 37 °C for the indicated time. Decreased expression of HER3 and pAkt was observed after either 1-h or 3-h incubation, suggesting an effect on signal transduction by HER3-VIA treatment (Fig. 6b). In NOD.CB17-*Prkdc*^*scid*^/J mice bearing MDA-MB-468, HER3-VIA slowed tumor growth (Fig. 6c) when compared to controls (*p* < 0.001). We also analyzed HER3 expression and signaling in the MDA-MB-468 tumors treated with HER3-VIA. Again, HER3-VIA treatment was associated with a decrease in HER3 and pHER3 expression as analyzed by western blot (Fig. 6d**).** These data confirm that the HER3-VIA downregulates HER3 expression in both HER2+ and TNBC. However, there was no clear decrease in pAkt (S473) or pErk1/2. These data suggest a complex effect of the polyclonal antibodies on downstream signaling from the HER3 receptor, which may differ depending on the tumor subtype targeted.

## Discussion

Although EGFR and HER2-targeted therapy has substantial activity in EGFR and HER2-overexpressing malignancies, respectively, therapeutic resistance and progression eventually develops in responders with metastatic disease who remain on therapy. Significant data implicate HER3 and more specifically, EGFR:HER3 and HER2:HER3 heterodimers as mediators of this resistance [36]. Monoclonal antibodies would be one mechanism for inhibiting HER family heterodimerization, but trastuzumab is ineffective against HER2:HER3 heterodimers [37], resistance develops to trastuzumab/pertuzumab combinations, there are no pertuzumab equivalents for EGFR, and anti-HER3 monoclonal antibodies are thus far not commercially available [38]. Our overall goal has been to develop an immunologic strategy to target HER3-expressing malignancies, reasoning that immune responses will be adaptive, pleiotropic, and persistent. Previously, we generated an Ad-HER3 vaccine and demonstrated that it induced potent HER3-specific T cell responses with anti-tumor activity [20]. In that study, we also observed preliminary evidence of the induction of HER3-VIA polyclonal serum in Ad-HER3-vaccinated mice, which suggested that further characterization of the antibody response was warranted thus leading to the experiments presented herein. We believe that while the T cell response is often regarded as the primary anti-tumor effector mechanism, antibody responses are also important as major histocompatibility complex (MHC) downregulation and epitope loss, observed in some tumors, would render them resistant to T cell-mediated cell death [39].

Vaccine-induced antibodies by virtue of their polyclonality should have the capacity to bind to multiple different epitopes and have multiple different functions, including commonly ascribed immune activities such as ADCC and CDC. Indeed, in the current study we observed immune-mediated anti-tumor activity mediated by Ad-HER3-induced antibodies (CDC and ADCC against HER3-expressing HER2+ and TNBC cells).

Additional anti-tumor activities that may be mediated by antibodies include internalization and signaling inhibition of the target-surface-expressed molecule [40–42]. Indeed, we observed that the anti-proliferative effects of HER3-VIA were likely due to internalization and signaling inhibition rather than inhibition of heregulin binding of HER3. These findings are consistent with our previously reported ability to generate polyclonal antibodies against the HER family member HER2, where we observed that these antibodies mediated HER2 receptor internalization and degradation in both mouse and human studies [19].

An important implication of HER3 downregulation is inhibition of signaling through HER3 heterodimers. Interestingly, treatment of lapatinib-sensitive and lapatinib-resistant BT474 cells with HER3-VIA led to decreased HER3, pHER3 and pERK1/2 as expected, but only treatment of the resistant BT474 cell led to a decrease in HER2, pAkt(S473), pS6, p4EPB1, and survivin expression. The decrease in the protein survivin, an inhibitor of apoptosis, suggests that there is also an increase in apoptotic cells after Ad-HER3 treatment. In a previous study [43], when the partially trastuzumab-resistant 4 T1-HER2-expressing tumors were treated with lapatinib or HER2-VIA alone, we observed no change in survivin expression, but when these tumors were treated with a combination of lapatinib and HER2-VIA, we observed a decrease in survivin expression [27] implying that complete HER2 signaling blockade decreased survivin expression [43]. In an analogous fashion, our current findings suggest that complete blockade of HER2:HER3 signaling in lapatinib-refractory tumors is accomplished by treatment with HER3-VIA, resulting in the decreased expression of survivin.

An important observation was that the growth in vitro and in vivo of more aggressive breast cancer subtypes such as HER2+ breast cancer and TNBC, and of lapatinib-refractory (HER2 small molecule inhibitor-refractory) tumors was slowed by HER3-VIA. We believe our findings lay a framework for an immune-mediated strategy for treating aggressive breast cancer subtypes (TNBC and HER2+ breast cancer), by co-administration of Ad-HER3 with HER2-targeted or EGFR-targeted therapies. Also, HER3 may play a role in therapeutic resistance to anti-estrogen therapies in estrogen receptor (ER)-positive breast cancers [44–47], and the development of castration resistance in prostate cancers [48]. Therefore, our findings also suggest a role for immunization with Ad-HER3 to induce HER3-VIA prior to the development of therapeutic resistance. Clinical trials of these approaches are in development.

## Conclusions

In this study, we revealed that polyclonal antibodies induced by Ad-HER3 vaccine (HER3-VIAs) are multifunctional, including induction of CDC, ADCC, anti-proliferative effect, HER3 internalization, and interruption of HER3 heterodimer-driven tumor signaling pathways. In addition to the T cell anti-tumor response induced by Ad-HER3, the HER3-VIAs provide additional functions to eliminate tumors in which HER3 signaling mediates aggressive behavior or acquired resistance to HER2-targeted therapy. These data support clinical studies of vaccination against HER3 prior to or concomitantly with other therapies to prevent outgrowth of therapy-resistant HER2+ and triple negative clones.

## Additional files

Additional file 1: **Figure S1.** Flow cytometric detection of HER3 on human breast cancer cell lines with anti-HER3 monoclonal antibodies (mAb) and HER3-VIA. To detect HER3-specific antibodies that could recognize membrane-associated HER3, binding of HER3-VIA in mouse serum was tested by flow cytometry against a series of human HER3-expressing breast tumor cell lines as targets, including the high HER3-expressing BT474, BT474M1, SKBR3, and T47D, and the HER3 negative, triple negative MDA-MB-231 tumor cell lines. Upper histograms: cells were stained with commercially available PE-conjugated anti-HER3 mAb (filled histogram), or PE-conjugate isotype control IgG as a negative staining (open histogram). Lower histograms: cells were incubated with HER3-VIA or GFP-VIA (1:100 dilution in medium), followed by staining with PE-conjugated anti-mouse IgG mAb. Filled histogram, HER3-VIA; open histogram, GFP-VIA. Percentage of staining positive cells and median fluorescence intensity are shown in each histogram. (PDF 119 kb)

Additional file 2: **Table S1.** Epitope mapping of HER3-VIA using spotted 15-mer peptide arrays. Epitope mapping was performed using spotted peptide arrays of 15-mer peptides overlapping by four amino acids representing the full length of the human HER3 protein. HER3 peptides were coated onto cellulose membranes using a Spot Robot ASP 222 (AbiMed) and epitope mapping of HER3-VIA (1:100 dilution in saline) was performed as described [26]. (PDF 36 kb)

Additional file 3: **Figure S2.** Flow cytometric detection of cell surface EGFR/HER2/HER3 expression after treatment with HER3-VIA, trastuzumab, cetuximab and lapatinib. To assess the internalization of EGFR family receptors, SKBR3 cells, positive for EGFR/HER2/HER3, were incubated with HER3-VIA, GFP-VIA (1:100 dilution), trastuzumab (1 μM), cetuximab (1 μM), or lapatinib (1 μM) for 3 h or 24 h. Cells were harvested using cell-dissociation buffer and stained with PE-conjugated anti-EGFP (clone 5E10D3, Novus Biologicals), anti-HER2 (clone Neu 24.7, BD Bioscience) or anti-HER3 antibody (Clone 1B4C3, BioLegend) and acquired by LSRII flow cytometer. Isotype control mouse IgG was used as negative control staining and is shown as filled gray histograms. Experiments were performed four times for EGFR expression analysis and twice for HER2 and HER3, and representative histograms are shown. Median fluorescence intensities (MFIs) of reagent-treated cells were compared to untreated control cells and ratios (MFI of treated/MFI of untreated) were calculated in each experiment. The averages of ratios are shown in each histogram. (PDF 732 kb)

## Abbreviations

ADCC: Antibody-dependent cellular cytotoxicity
Ad-HER3: Adenovirus encoding full length human HER3
APC: Allophycocyanin
BSA: Bovine serum albumin
CDC: Complement-dependent cytotoxicity
CMV: Cytomegalovirus
CNS: Central nervous system
DAPI: 4′,6-Diamidino-2-phenylindole
DMEM: Dulbecco’s modified Eagle’s medium
EGFR: Epidermal growth factor receptor
ELISA: Enzyme-linked immunosorbent assay
FBS: Fetal bovine serum
GFP: Green fluorescence protein
HER2: Human epidermal growth factor receptor 2
HER3: Human epidermal growth factor receptor 3
IACUC: Institutional Animal Care and Use Committee
mAb: Monoclonal antibodies
MHC: Major histocompatibility complex
MTT: 3-(4,5-Dimethylthiazol-2-yl)-2.5-diphenyltetrazolium bromide
nIR: Near infrared
PBS: Phosphate-buffered saline
PCR: Polymerase chain reaction
PE: Phycoerythrin
PFA: Paraformaldehyde
SCID: Severe combined immunodeficiency
TKI: Tyrosine kinase inhibitor
TNBC: Triple negative breast cancer
VIA: Vaccine-induced antibody
