# Supplementary figures and images for: Polyfunctional anti-human epidermal growth factor receptor 3 (anti-HER3) antibodies induced by HER3 vaccines have multiple mechanisms of antitumor activity against therapy resistant and triple negative breast cancers
Source: Breast Cancer Res. 2018 Aug 9;20:90. doi: 10.1186/s13058-018-1023-x (PMC6085609; doi:10.1186/s13058-018-1023-x)

**anti-HER3 mAb  
vs.  
Control IgG**

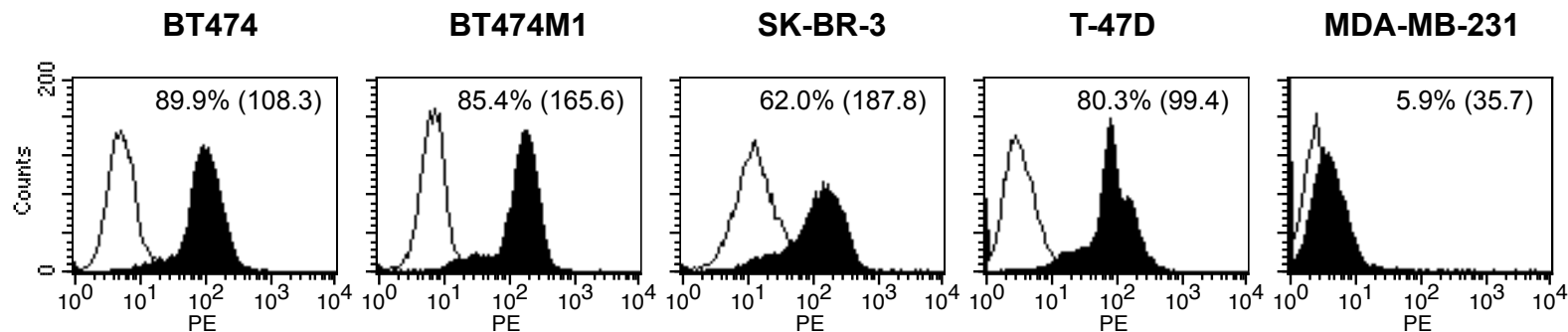

**HER3-VIA  
vs.  
GFP-VIA**

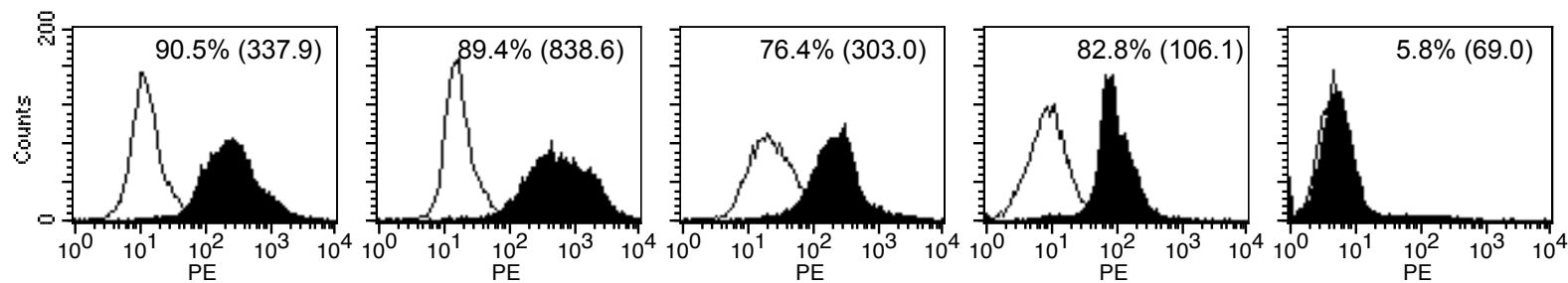

Supplement: Supplementary file 1 — Figure S1. Flow cytometric detection of HER3 on human breast cancer cell lines with anti-HER3 monoclonal antibodies (mAb) and HER3-VIA. To detect HER3-specific antibodies that could recognize membrane-associated HER3, binding of HER3-VIA in mouse serum was tested by flow cytometry against a series of human HER3-expressing breast tumor cell lines as targets, including the high HER3-expressing BT474, BT474M1, SKBR3, and T47D, and the HER3 negative, triple negative MDA-MB-231 tumor cell lines. Upper histograms: cells were stained with commercially available PE-conjugated anti-HER3 mAb (filled histogram), or PE-conjugate isotype control IgG as a negative staining (open histogram). Lower histograms: cells were incubated with HER3-VIA or GFP-VIA (1:100 dilution in medium), followed by staining with PE-conjugated anti-mouse IgG mAb. Filled histogram, HER3-VIA; open histogram, GFP-VIA. Percentage of staining positive cells and median fluorescence intensity are shown in each histogram. (PDF 119 kb) [file 13058_2018_1023_MOESM1_ESM.pdf]

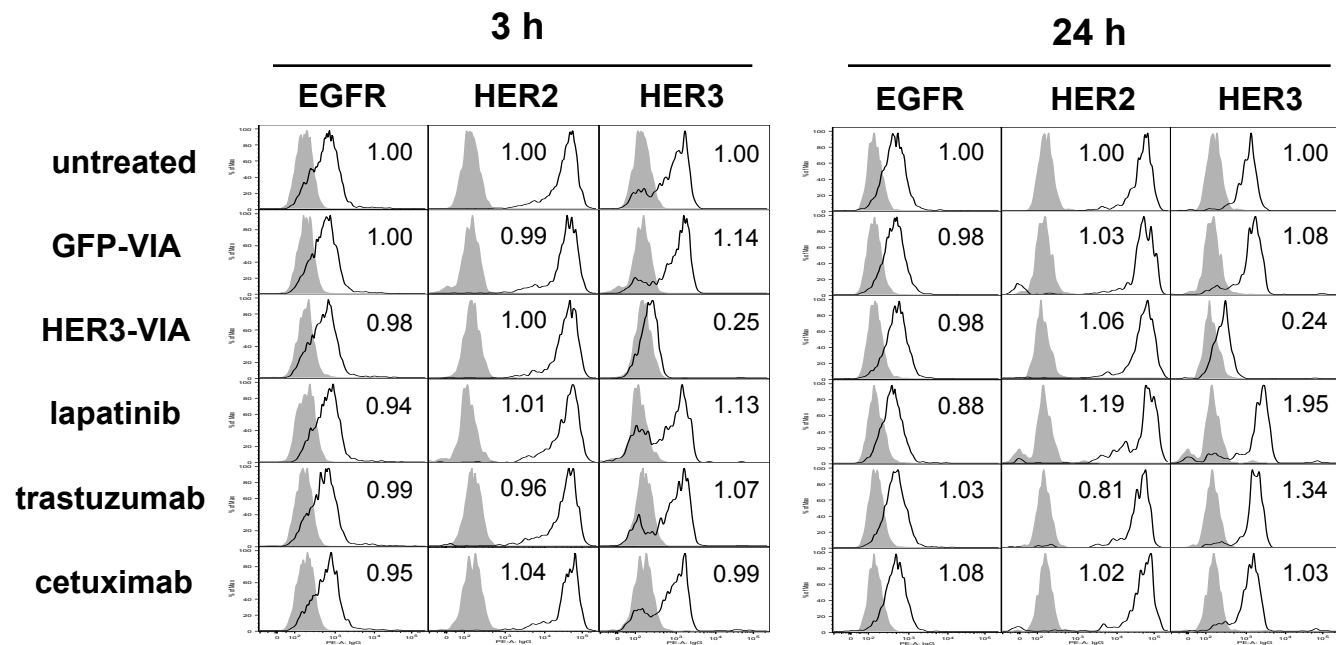

Supplement: Supplementary file 3 — Figure S2. Flow cytometric detection of cell surface EGFR/HER2/HER3 expression after treatment with HER3-VIA, trastuzumab, cetuximab and lapatinib. To assess the internalization of EGFR family receptors, SKBR3 cells, positive for EGFR/HER2/HER3, were incubated with HER3-VIA, GFP-VIA (1:100 dilution), trastuzumab (1 μM), cetuximab (1 μM), or lapatinib (1 μM) for 3 h or 24 h. Cells were harvested using cell-dissociation buffer and stained with PE-conjugated anti-EGFP (clone 5E10D3, Novus Biologicals), anti-HER2 (clone Neu 24.7, BD Bioscience) or anti-HER3 antibody (Clone 1B4C3, BioLegend) and acquired by LSRII flow cytometer. Isotype control mouse IgG was used as negative control staining and is shown as filled gray histograms. Experiments were performed four times for EGFR expression analysis and twice for HER2 and HER3, and representative histograms are shown. Median fluorescence intensities (MFIs) of reagent-treated cells were compared to untreated control cells and ratios (MFI of treated/MFI of untreated) were calculated in each experiment. The averages of ratios are shown in each histogram. (PDF 732 kb) [file 13058_2018_1023_MOESM3_ESM.pdf]
