# Supplementary material for: Polyfunctional anti-human epidermal growth factor receptor 3 (anti-HER3) antibodies induced by HER3 vaccines have multiple mechanisms of antitumor activity against therapy resistant and triple negative breast cancers
Source: Breast Cancer Res. 2018 Aug 9;20:90. doi: 10.1186/s13058-018-1023-x (PMC6085609; doi:10.1186/s13058-018-1023-x)
Supplement: Supplementary file 2 — Table S1. Epitope mapping of HER3-VIA using spotted 15-mer peptide arrays. Epitope mapping was performed using spotted peptide arrays of 15-mer peptides overlapping by four amino acids representing the full length of the human HER3 protein. HER3 peptides were coated onto cellulose membranes using a Spot Robot ASP 222 (AbiMed) and epitope mapping of HER3-VIA (1:100 dilution in saline) was performed as described [26]. (PDF 36 kb) [file 13058_2018_1023_MOESM2_ESM.pdf]

Protein Region	Epitope(s) Position	Amino Acid Sequence
ECD	101-111	nlrvvrgtqvy
ECD	153-167	dklchmdtidwrdiv
ECD	185-191	chevck
ECD	209-223	icapqcnghcfgpnp
ECD	369-375	ngdpwhk
ECD	501-511	dplcssggcwg
ECD	589-599	cphgvlgakgp
TM	649-663	iaglvvifmmlggtf
ICD	681-691	lergesiepld
ICD	865-875	qllyseaktpi
ICD	881-895	esihfgkythqsdvw
ICD	901-915	vwelmtfgaepyagl
ICD	981-988	esgpgiap
ICD	1037-1051	tlnrprgsqsllsps
ICD	1105-1119	eaelqekvsmcrsrs
ICD	1153-1163	eedvngyvmpd
ICD	1249-1255	scplhp
ICD	1268-1279	dgggpggdyaam
